# Nanopore-based direct sequencing of RNA transcripts with 10 different modified nucleotides reveals gaps in existing technology

**DOI:** 10.1093/g3journal/jkad200

**Published:** 2023-09-01

**Authors:** Joshua T Burdick, Annelise Comai, Alan Bruzel, Guangxin Sun, Peter C Dedon, Vivian G Cheung

**Affiliations:** Life Sciences Institute, University of Michigan, Ann Arbor, MI 48109, USA; Department of Pediatrics, University of Michigan, Ann Arbor, MI 48109, USA; Life Sciences Institute, University of Michigan, Ann Arbor, MI 48109, USA; Department of Pediatrics, University of Michigan, Ann Arbor, MI 48109, USA; Life Sciences Institute, University of Michigan, Ann Arbor, MI 48109, USA; Department of Pediatrics, University of Michigan, Ann Arbor, MI 48109, USA; Department of Biological Engineering, Massachusetts Institute of Technology, Cambridge, MA 02139, USA; Department of Biological Engineering, Massachusetts Institute of Technology, Cambridge, MA 02139, USA; Life Sciences Institute, University of Michigan, Ann Arbor, MI 48109, USA; Department of Pediatrics, University of Michigan, Ann Arbor, MI 48109, USA

**Keywords:** RNA sequencing, Nanopore, chemical modification of RNA

## Abstract

RNA undergoes complex posttranscriptional processing including chemical modifications of the nucleotides. The resultant-modified nucleotides are an integral part of RNA sequences that must be considered in studying the biology of RNA and in the design of RNA therapeutics. However, the current “RNA-sequencing” methods primarily sequence complementary DNA rather than RNA itself, which means that the modifications present in RNA are not captured in the sequencing results. Emerging direct RNA-sequencing technologies, such as those offered by Oxford Nanopore, aim to address this limitation. In this study, we synthesized and used Nanopore technology to sequence RNA transcripts consisting of canonical nucleotides and 10 different modifications in various concentrations. The results show that direct RNA sequencing still has a baseline error rate of >10%, and although some modifications can be detected, many remain unidentified. Thus, there is a need to develop sequencing technologies and analysis methods that can comprehensively capture the total complexity of RNA. The RNA sequences obtained through this project are made available for benchmarking analysis methods.

## Introduction

RNA is made up of 4 nitrogenous bases (adenine, cytosine, guanine, and uracil) and a sugar-phosphate backbone. The bases and sugar of RNA undergo chemical alteration, leading to >150 modified nucleotides (Machnicka *et al.* 2013; Boccaletto *et al.* 2018). Some are simple modifications such as the addition of a methyl group in *N*6-methyladenosine (m6A), and others are complex multistep modifications, resulting in wybutosine (Noma *et al.* 2006) and queuosine (Kuchino *et al.* 1976). These modifications are an essential component of the regulatory code of cells. They determine the structure, stability, and function of RNA (Byszewska *et al.* 2014; Haag *et al.* 2016; Liu *et al.* 2016; Nakano *et al.* 2016; Kawarada *et al.* 2017; Galloway and Cowling 2019; Lan *et al.* 2019; Huang *et al.* 2020).

RNA sequences were once assumed to be nearly identical to the underlying DNA sequences except for the occasional RNA editing by adenosine and cytidine deaminases (Powell *et al.* 1987; Bass and Weintraub 1988; Bass 2002). Although we have realized the inaccuracy of this assumption, we still study RNA and design RNA therapeutics, as if RNA sequences are merely identical to their corresponding DNA. This approach leads to an incomplete understanding of RNA and ineffective therapeutics. The extent of chemical modifications varies depending on the RNA and cellular conditions. In transfer RNA, about 10–20% of the bases or sugar are modified (Jühling *et al.* 2009; Boccaletto *et al.* 2018; Suzuki 2021), and in messenger RNA, there are also many types of modifications including half of the transcripts having at least one m6A (Dominissini *et al.* 2012; Ke *et al.* 2017). The modifications in tRNAs affect protein synthesis; for example, the ribonucleotide at position 34 in the anticodon is often modified. These modifications facilitate wobble pairing, thus allowing more codons to be deciphered by tRNAs (Suzuki 2021). The modifications in mRNA are also functional, and they affect RNA processing and transcript stability. They influence how single-stranded RNA forms hairpins and the interactions between RNA and RNA as well as between RNA and DNA. Additionally, modifications influence the interactions of RNA with processing proteins, for example, pseudouridine (Y) enhances splicing and is found to be enriched in splice sites and splicing regulatory elements to promote alternative splicing (Martinez *et al.* 2022). Similarly, m6A affects splicing by interacting with hnRNPG, which influences RNA polymerase II occupancy and promotes exon inclusion (Zhou *et al.* 2019). Our recent study found that RNA abasic sites stabilize R-loops and pause RNA polymerase II elongation in non-coding RNAs (Watts *et al.* 2022). While we are beginning to decipher how the modifications individually influence the structure and function of RNA, we do not know their combinatorial impact. The lack of methods to sequence RNA with all the modifications hampers the study of modifications in sequence context. To gain a full understanding of how RNA regulates cell function and to develop precise RNA therapeutics that are potent and efficacious require knowing the complete sequences of the RNA transcripts, including carrying out all modifications at single-molecule resolution.

RNA-sequencing technologies are underdeveloped. The so-called RNA-sequencing (“RNA-seq”) methods do not sequence RNA, rather RNA is converted back to DNA and the resultant DNA is sequenced; in the reverse transcription, all modifications are lost (Alfonzo *et al.* 2021). Ways to directly sequence RNA as single molecules are beginning to emerge, the main one being that developed by Oxford Nanopore Technologies (henceforth referred to as Nanopore). In Nanopore sequencing, RNA molecules are pulled through nano-sized pores. As they pass through these pores, the distinct ribonucleotides generate unique current patterns. In the current version of the Oxford Nanopore device (R9), about 5 nucleotides pass through the narrowest part of the pores, known as the reader-head, at a given time (Garalde *et al.* 2018; Li *et al.* 2018; Leger *et al.* 2021). Because the reader-head accommodates ∼5 nucleotides, the sequencing resolution has not yet reached the level of single-nucleotide precision.

To translate the current patterns into sequences encompassing the 4 canonical nucleotides (A, C, G, and U) and the >150 modifications, it is necessary to train base-calling algorithms to establish the correspondence between the nucleotides and their corresponding current patterns. Many groups have developed methods to convert the current signals to sequence (Cozzuto *et al.* 2020; Jenjaroenpun *et al.* 2021; Leger *et al.* 2021; Liu *et al.* 2021; Abebe *et al.* 2022; Hendra *et al.* 2022; Piechotta *et al.* 2022; Stephenson *et al.* 2022). But today, none of the base-calling algorithms can decipher the total complexity of RNA; most of them can only identify the canonical nucleosides plus a few modified nucleosides, namely m6A and Y.

To advance to a comprehensive RNA-sequencing capability, it is crucial to assess the current state of the technology. Here, we synthesized a set of RNA transcripts with only canonical nucleotides and with 10 modifications in different proportions. The RNAs were sequenced using the MinION from Oxford Nanopore to provide a resource for algorithm development. We carried out basic analysis including assessing the accuracy of Nanopore direct RNA sequencing and determining what modifications could be recognized in the RNA. We found that the substitution error rate of RNA transcripts with even just the canonical nucleosides is >10%, and while some modified nucleosides such as Ys and some of the modified cytosines can be identified, others are not. As base-calling algorithms are developed, this set of sequences is available for benchmarking.

## Materials and methods

### RNA preparation

The genomic region chr19:45406985–45408892 (on genome build hg19) was cloned from human fibroblast DNA into the pcDNA3.1-GFP(1–10) (Addgene cat# 70219) vector. The clone sequence was as follows:

UUGAGCAUUUCAUCCGGAGUCUGGCCGCCCUGACCUUCCC
CCAGCCGCCUGCAGGGGGCGCCAGAGGGCCGGAGCACGGA
AAGCAGCGGAUCCUUGAUGCUGCCUUAAGUCCGGCUCAGA
GGGGCGCAGCGUGGCCUGGGGUCGCUAUCUUCCCAUCCGG
AACAUCUGCCCUGCUGGGGGACACUACGGGCCUUCCCUUG
CCUGAGGGUAGGGUCUCAAGGUCACUUGCCCCCAGCUUGA
CCUGGCCGGAGUGGCUAUAGAGGACUUUGUCCCUGCAGAC
UGCAGCAGCAGAGAUGACACUGUCUCUGAGUGCAGAGAUG
GGGGCAGGGAGCUGGGAGAGGGUUCAAGCUACUGGAACAG
CUUCAGAACAACUAGGGUACUAGGAACUGCUGUGUCAGGG
AGAAGGGGCUCAAGGACUCGCAGGCCUGGGAGGAGGGGCC
UAGGCCAGCCAUGGGAGUUGGGUCACCUGUGUCUGAGGAC
UUGGUGCUGUCUGGAUUUUGCCAACCUAGGGCUGGGGUCA
GCUGAUGCCCACCACGACUCCCGAGCCUCCAGGAACUGAA
ACCCUGUCUGCCCCCAGGGUCUGGGGAAGGAGGCUGCUGA
GUAGAACCAACCCCAGGUUACCAACCCCACCUCAGCCACC
CCUUGCCAGCCAAAGCAAACAGGCCCGGCCCGGCACUGGG
GGUUCCUUCUCGAACCAGGAGUUCAGCCUCCCCUGACCCG
CAGAAUCUUCUGAUCCCACCCGCUCCAGGAGCCAGGAAUG
AGUCCCAGUCUCUCCCAGUUCUCACUGUGUGGUUUUGCCA
UUCAUCUUGCUGCUGAACCACGGGUUUCUCCUCUGAAACA
UCUGGGAUUUAUAACAGGGCUUAGGAAAGUGACAGCGUCU
GAGCGUUCACUGUGGCCUGUCCAUUGCUAGCCCUAACAUA
GGACCGCUGUGUGCCAGGGCUGUCCUCCAUGCUCAAUACA
CGUUAGCUUGUCACCAAACAUACCCGUGCCGCUGCUUUCC
CAGUCUGAUGAGCAAAGGAACUUGAUGCUCAGAGAGGACA
AGUCAUUUGCCCAAGGUCACACAGCUGGCAACUGGCAGAG
CCAGGAUUCACGCCCUGGCAAUUUGACUCCAGAAUCCUAA
CCUUAACCCAGAAGCACGGCUUCAAGCCCCUGGAAACCAC
AAUACCUGUGGCAGCCAGGGGGAGGUGCUGGAAUCUCAUU
UCACAUGUGGGGAGGGGGCUCCCCUGUGCUCAAGGUCACA
ACCAAAGAGGAAGCUGUGAUUAAAACCCAGGUCCCAUUUG
CAAAGCCUCGACUUUUAGCAGGUGCAUCAUACUGUUCCCA
CCCCUCCCAUCCCACUUCUGUCCAGCCGCCUAGCCCCACU
UUCUUUUUUUUCUUUUUUUGAGACAGUCUCCCUCUUGCUG
AGGCUGGAGUGCAGUGGCGAGAUCUCGGCUCACUGUAACC
UCCGCCUCCCGGGUUCAAGCGAUUCUCCUGCCUCAGCCUC
CCAAGUAGCUAGGAUUACAGGCGCCCGCCACCACGCCUGG
CUAACUUUUGUAUUUUUAGUAGAGAUGGGGUUUCACCAUG
UUGGCCAGGCUGGUCUCAAACUCCUGACCUUAAGUGAUUC
GCCCACUGUGGCCUCCCAAAGUGCUGGGAUUACAGGCGUG
AGCUACCGCCCCCAGCCCCUCCCAUCCCACUUCUGUCCAG
CCCCCUAGCCCUACUUUCUUUCUGGGAUCCAGGAGUCCAG
AUCCCCAGCCCCCUCUCCAGAUUACAUUCAUCCAGGCACA
GGAAAGGACAGGGUCAGGAAAGGAGGACUCUGGGCGGCAG
CCUCCACAUUCCCCUUCCACGCUUGGCCCCCAGAAUGGAG
GAGGGUGUCUGGAUUACUGGGCGAGGUGUCCUCCCUUCCU
GGGGACUGUGGGGGGUGGUCAAAAGACC

The sequence includes 691 distinct 5-mers, about 70% [691/(4^5^)] of the 5-mers of the RNA synthesized with just the canonical nucleotides.

We used the MEGAscript T7 transcription kit (ThermoFisher, cat# AM1334) for in vitro transcription (IVT) in the presence of varying amounts of modified nucleotides to prepare RNA for Nanopore sequencing. The modified nucleotides used were the 2′-*O*-methyl-nucleotide set (TriLink Biotechnologies, cat# K-1012), *N*6-methyladenosine-5′-triphosphate (TriLink Biotechnologies, cat# N-1013-1), *N*1-methyladenosine-5′-triphosphate (TriLink Biotechnologies, cat# N-1042-1), 5-methylcytidine-5′-triphosphate (TriLink Biotechnologies, cat# N-1014-1), 5-hydroxymethylcytidine-5′-triphosphate (TriLink Biotechnologies, cat# N-1087-1), pseudouridine-5′-triphosphate (TriLink Biotechnologies, cat# N-1019-1), and biotin-11-CTP (Perkin-Elmer, cat# NEL542001EA). In vitro-transcribed RNAs were purified from their reaction mixes using RNAClean XP beads (Beckman Coulter, cat# A63987), and the integrity of the RNA (∼2 kb, and no evidence of degradation) was verified using the Agilent RNA 6000 Nano Kit (cat# 5067-1511).

### Identification of biotinylated cytidine

An RNA dot blot of the biotin-C IVT RNA with canonical IVT RNA as carrier was prepared. After UV crosslinking the RNA spots to an Amersham Hybond-N^+^ membrane (cat# RPN119B), the ThermoFisher chemiluminescent nucleic acid detection module kit (cat# 89880) was used to visualize the biotinylated RNA on X-ray film. Then, ImageJ was used to quantitate the intensity of the spots.

### Mass spectrometry

RNA from each sample (2 µg) was hydrolyzed in a 50-µL digestion cocktail containing 8 U benzonase, 5 U calf intestinal alkaline phosphatase, 0.15 U phosphodiesterase I, 0.1 mM deferoxamine, 0.1 mM butylated hydroxytoluene, 5 ng coformycin, 50 nM internal standard [^15^N]_5_-deoxyadenosine, 2.5 mM MgCl_2_, and 5 mM Tris-HCl buffer pH 8.0. The digestion mixture was incubated at 37°C for 6 h.

After digestion, all 9 samples were analyzed by chromatography-coupled triple-quadrupole mass spectrometry (LC-MS/MS). For each sample, 600 ng of hydrolysate was injected into each of 3 technical replicates. Using synthetic standards, HPLC retention times of RNA modifications were confirmed on a Waters Acuity BEH C18 column (50 × 2.1 mm inner diameter, 1.7 µm particle size) coupled to an Agilent 1290 HPLC system and an Agilent 6495 triple-quadrupole mass spectrometer. The HPLC system was operated at 25°C and a flow rate of 0.3 mL/min in a gradient [Supplementary Table 1 with buffer A (0.02% formic acid in water) and buffer B (0.02% formic acid in 70% acetonitrile)]. The HPLC column was coupled to the mass spectrometer with an electrospray ionization source in positive mode with the following parameters: dry gas temperature, 200°C; gas flow, 11 L/min; nebulizer, 20 psi; sheath gas temperature, 300°C; sheath gas flow, 12 L/min; capillary voltage, 3,000 V; nozzle voltage, 0 V. Multiple reaction monitoring mode was used for the detection of product ions derived from the precursor ions for all the RNA modifications with instrument parameters, including the collision energy optimized for maximal sensitivity for the modification. Based on synthetic standards (Biosynth) with optimized collision energies, the following transitions and retention times were monitored: *N*1-methyladenosine (m1A), *m/z* 282 → 150, 1.2 min; m6A, *m/z* 282 → 150, 8.7 min; 5-hydroxymethylcytidine (hm5C), *m/z* 274.1 → 142.1, 0.88 min; Y, *m/z* 245 → 191, 0.88 min. Signal intensities for each ribonucleoside were normalized by dividing by the sum of the UV signal intensities of the 4 canonical ribonucleosides recorded with an in-line UV spectrophotometer at 260 nm.

### Nanopore sequencing

In-vitro-transcribed RNAs were 3′-polyadenylated using Oxford Nanopore's protocol for polyadenylating with *Escherichia coli* poly(A) polymerase, followed by Oxford Nanopore's protocol for direct RNA sequencing (SQK-RNA002). RNA samples were sequenced using an Oxford Nanopore MinION Mk1B device or a 5-cell GridION sequencer, with FLO-MIN106D flow cells (which use the v9.4.1 pore that holds 5 nucleotides at a time (Li *et al.* 2018; Zeng *et al.* 2020; Leger *et al.* 2021)). No differences were noted in the quality of the sequences from the MinION or GridION.

### Base-calling and aligning

Base-calling was performed using Guppy v6.3.4 with the rna_r9.4.1_70bps_hac.cfg model (which is optimized for accuracy over speed). Reads were aligned to the clone sequence using minimap2 v2.17 (Li 2018), using the “-x map-ont” preset. This preset disables splice junction search, and requires a score >0, assuming matching score = 2, mismatching penalty = 4, gap open penalty = 4, and gap extension penalty = 2.

### Error rates

In each sample, at each nucleotide position, we compared the RNA sequence to the corresponding sequence of the DNA sense strand, and then, we calculated the error rate as a ratio of mismatches to the total sequence reads at position (matched + mismatched). Those ratios are presented as percentages in the figures.

For each sample, we also break down the errors by the 4 nucleotides in the DNA template. For each of the 4 (A, C, G, and T) nucleotides in the DNA, we looked for the number of mismatches in the RNA. In each sample, for each nucleotide, we determined the ratio of the number of mismatches to the total number of reads for the nucleotides and reported the ratios in percentages as A-to-X, C-to-X, G-to-X, and U-to-X where X is A, C, G, and U from Nanopore. The error rates calculated as implemented in our Python scripts are the same as those from EpiNano.

### Dwell times

To compute dwell times, reads were first resquiggled using f5c version 0.8 (Gamaarachchi *et al.* 2020), with the “—collapse-events” and “—signal-index” options. This computes the time interval at which each 5-base “event” went through the nanopore; dwell times were computed from those event bounds.

### Epinano

Epinano v1.2 (Liu *et al.* 2021) was used to estimate which bases were modified, which use levels of base-calling errors: mismatches, insertions, deletions, and base qualities. We ran “Epinano_Variants.py –type t” to specify alignment to the sequenced transcriptome sequence; apart from this, we used default arguments.

### Tombo

Tombo v1.5 (Stoiber *et al.* 2017) was used to identify modified sites. We used its “model_sample_compare” mode, which builds a model of canonical (unmodified) squiggles. It then compares observed squiggles to this canonical model, computing a per-base score.

### Workflow automation

We used the Master of Pores, v2 (Cozzuto *et al.* 2020) pipeline to automate running several of these programs, on all of the samples. Specifically, we used the “mop_preprocess” module to run Guppy for base-calling, and minimap2 for alignment. We then used the “mop_mod” module to run Epinano and Tombo for modification calling.

### Principal component analysis

Principal component analysis (PCA) was performed by using 7 variables (A-to-X, C-to-X, G-to-X, U-to-X, insertion rates, deletion rates, and dwell time) for the 45 samples (the 10 samples with canonical nucleotides were combined into 1 sample). The data were normalized by using the *Z*-score. The PCA was calculated as the total variance explained. The principal component 1 (PC1) and principal component 1 (PC2) scores are plotted.

We also carried out a PCA with up to 5,000 randomly selected reads from each sample and the current and dwell times for the 5-mers that are most different among the modifications were used as variables. To select the variables for the PCA, for each 5-mer in our data, we performed ANOVA to identify the current signals that are most variable among the samples vs within, the top 5% of the most variable current signals were used as variables. The same analysis was used to identify the dwell times for the PCA. The variables were normalized by *Z*-score. The PCA was calculated as the total variance explained.

Another PCA was performed for each sample with 7 variables (A-to-X, C-to-X, G-to-X, U-to-X, insertion rates, deletion rates, and dwell time) for each nucleotide. The data were also normalized by *Z*-score. The PCA was calculated as the total variance explained. For each sample, we then looked for the sites with the most modified nucleotides based on the PC1 and PC2 scores. We identified the outlier sites as those with PC scores furthest by Euclidean distance from the center of the plots; then at those locations, in each read, we looked for errors and longer dwell times to identify the most likely modified nucleotides. For the m6A, biotinylated-C, and Y samples, we only looked at the A, C, and U, respectively.

## Results

### Synthesis and sequencing of RNA transcripts

We synthesized a set of RNA transcripts with only canonical nucleotides and also with 10 different modified nucleotides. We then performed direct RNA sequencing using the Oxford Nanopore technology on those 2 kb RNA transcripts.

Some of the IVT reactions included canonical nucleotides only, others included different proportions of a modified base or sugar nucleotide and the corresponding canonical nucleotide. The modified bases consist of m1A, m6A, biotinylated cytidine, hm5C, 5-methylcytidine (m5C), Y, and the modified sugars were 2′-*O*-methyladenosine (Am), 2′-*O*-methylcytidine (Cm), 2′-*O*-methylguanosine (Gm), and 2′-*O*-methyluridine (Um). Each of the modified nucleotides was added with its corresponding canonical nucleotide in 1:100, 1:10, 1:5, and 1:2 proportions (modified nucleotide:canonical nucleotide).

**Fig. 1.**
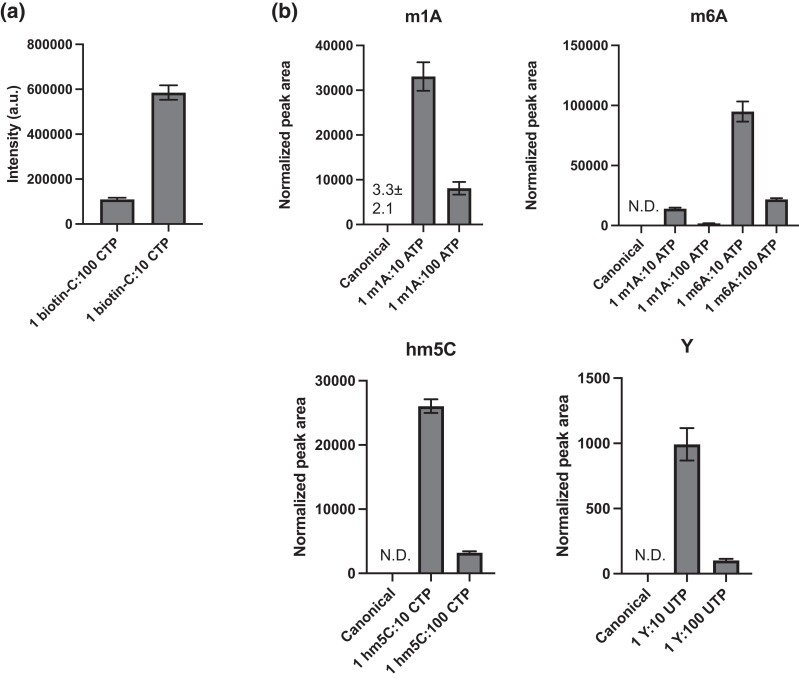
Validation of the modified nucleotide in the RNA transcripts. a) Quantification by the dot blot of biotin-C incorporation into transcripts, for 2 different concentrations of biotin-C added to the IVT reaction. b) Quantification by LC-MS/MS of modified ribonucleosides m1A, m6A, hm5C, and Y in the transcripts, for 2 different concentrations of each ribonucleoside triphosphate added to the IVT reaction. Data represent mean ± SD for 3 technical replicates.

To verify that the modified nucleotides were incorporated into the RNA transcripts, the samples were checked by chemiluminescent assay and LC-MS/MS. The samples with biotinylated cytidine were checked by chemiluminescence using streptavidin-horseradish peroxidase. Additionally, to obtain a more accurate assessment of the modifications, 9 samples were analyzed by mass spectrometry. These include 1 sample synthesized with just canonical nucleotides and samples with m1A, m6A, hm5C, and Y at 2 concentrations (1 part modified nucleotides to 10 parts canonical nucleotides, and 1 part modified to 100 parts canonical nucleotides). Figure 1a shows that the biotinylated cytidines were incorporated into the samples at about the expected proportions. The LC-MS/MS analysis also confirmed that m1A, m6A, hm5C, and Y were incorporated into the transcripts (Fig. 1b, Supplementary Table 2). The level of the modifications in each pair of modified RNA samples was expected to differ by 10-fold; the Y samples showed the expected 9.7-fold difference, and the m6A and hm5C showed 8-fold differences. As expected, there was contamination of the m1A samples with m6A from the spontaneous Dimroth rearrangement occurring at some point during transcript synthesis or processing (Wang *et al.* 2019).

All the synthesized RNA transcripts were polyadenylated, and then sequenced using the Oxford Nanopore MinION. The base-calling was carried out using Guppy, alignment was performed with Minimap2 (Li 2018), and sequence quality was assessed with Master of Pores 2 (Cozzuto *et al.* 2020; Supplementary Table 3).

### RNA sequences of transcripts synthesized with only canonical nucleotides

We started by analyzing the 10 independently synthesized and sequenced sets of RNA with just canonical nucleotides. The samples were sequenced deeply with an average coverage of 5,563 reads (median = 7,184 reads, range: 259–9,459 reads; see Table 1). The median PHRED (Ewing *et al.* 1998) score for the sequences was 17.5. In 7 of the 10 sets of samples, >90% of the reads have PHRED scores >15, in the other 3 sets, >82% of the reads have PHRED scores >15.

**Table 1.** RNA transcripts and their sequence coverage: RNA with only canonical nucleotides.

Samples	No. of reads	Samples	No. of reads
1	9,459	6	6,941
2	9,254	7	3,071
3	8,886	8	1,613
4	7,929	9	795
5	7,427	10	259

**Fig. 2.**
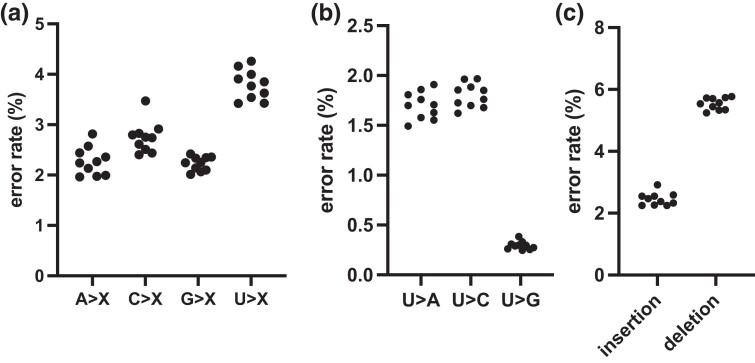
Sequence mismatches, insertions, and deletions in RNA sequences of transcripts with only canonical nucleotides. a) Transcript-wide error rate as the proportion of reads that are mismatched between DNA and RNA sequences. Data are shown for the 10 sets of independently synthesized and sequenced RNA transcripts, where each sample is shown as a dot. For example, A-to-X indicates that the sense strand DNA sequence was adenosine (A) but the corresponding transcribed RNA sequence was cytidine (C), guanosine (G), or uridine (U) and therefore denoted collectively as X. b) Breakdown of U-to-X substitution rates in a); proportion of reads that are A, C, or G in the RNA sequences when the corresponding sense strand DNA sequences were T, and a U is expected in the transcribed RNA strand. c) Insertion and deletion rates in the samples.

We then assessed the accuracy of RNA sequences obtained from transcripts composed of only canonical nucleotides. For these transcripts, the 2 main types of errors that contribute to mismatches between the RNA sequences and the corresponding DNA are errors from T7 RNA polymerase and errors from Oxford Nanopore sequencing. T7 RNA polymerase misincorporates about 1 base per 100,000 bases (error rate 10^−5^) and is prone to slippage in particular when transcribing A/T homopolymers (Chamberlin and Ring 1973; Huang *et al.* 2000; Nakano *et al.* 2012; Wons *et al.* 2015; Long *et al.* 2019). With that in mind, we calculated errors by comparing the sequence of the DNA sense strand with that of the transcribed RNA. The average substitution error rate across the 10 samples is 11.2% (median = 11.3, 10–13%). The results are highly consistent across the samples; Fig. 2a shows errors by types (A-to-X, C-to-X, G-to-X, and U-to-X, where X is A, C, G, or U) in each of the samples. Uridines were most prone to errors; their sequences were mostly misrepresented as adenosine and cytidine (U-to-A and U-to-C, Fig. 2b). There were also insertions and deletions (see Fig. 2c). The average insertion rate is 2.5% (median = 2.4%, 2.3–2.9%) and the average deletion rate is higher at 5.5% (median = 5.6%, 5.2–5.8%). Among those, 27.8% of insertions and 44.6% of deletions occurred following homopolymer runs of at least 3 bases. Others have also found a high rate of insertions and deletions following homopolymers in Nanopore sequencing (Gilpatrick *et al.* 2020; Delahaye and Nicolas 2021). In these homopolymers, cytidine was the most frequent base (at 47.6% of insertions, and 39.0% of deletions).

### RNA sequences of transcripts synthesized with canonical nucleotides plus modified nucleotides

Next, we turned to the RNA transcripts in vitro transcribed with 6 different base-modified nucleotides plus their corresponding canonical nucleotides. All the samples were deeply sequenced (median = 6,906 reads, range = 307–11,353 reads, Table 2). The sequences for some of the RNAs with modified bases have lower PHRED scores. While the sequence reads of the RNAs with only canonical bases have a median PHRED score of 17.5, those for the RNA with biotinylated cytidine and m5C are only 15 and those for 5hmC are 16. The sequences for transcripts with m1A and m6A have similar PHRED scores as the samples with only canonical nucleotides (median = 18), in all of the samples with m1A and m6A, 90% of the reads have PHRED scores of 15 or higher (for sequences of transcripts synthesized with 1 part modified base, 2 parts canonical base).

**Table 2.** RNA transcripts and their sequence coverage: RNA with modified bases.

Proportion	m1A	m6A	BiotinC	hm5C	m5C	Y
1:100	6,755	11,353	1,192	307	1,036	7,816
1:10	7,057	8,076	7,743	593	2,654	8,288
1:5	7,714	10,503	345	504	966	8,147
1:2	7,234	8,865	594	724	737	7,885

**Fig. 3.**
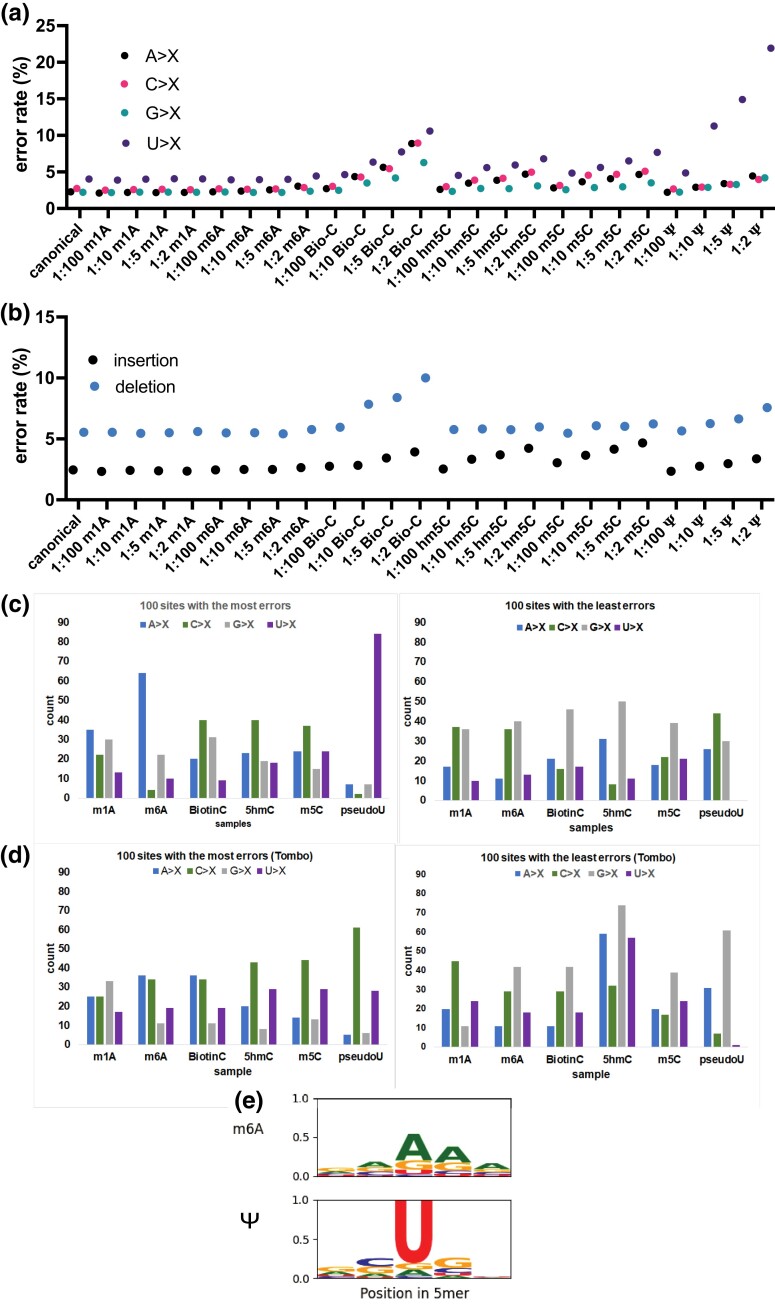
Sequence mismatches, insertions, and deletions in RNA sequences of transcripts synthesized with different modified nucleotides in various proportions. a) Mismatches between DNA and RNA sequences, as in Fig. 2a but for samples synthesized with different modified nucleobases in various proportions, and each sample is shown as a dot. b) Insertion and deletion rates, as in Fig. 2c. c and d) At the sites with the most errors [determined by error analysis c) and Tombo d)], the errors correspond to the modifications in the samples. Data from the sites with the least errors are shown as control. e) Sequence motifs of the 100 sites with the most errors in the RNA sequences of samples synthesized with m6A and Y, no sequence enrichment was found in other samples.

We then asked whether we could distinguish the modified nucleotides by various methods, including error analyses, the modified nucleotide prediction in Tombo (compare option; Stoiber *et al.* 2017), and the dwell time in the pores. Understandably, for the sequences of RNA with modified bases, the mismatches between the RNA and DNA sequences would reflect sequencing errors and misreadings of the modifications. Studies have also found that modifications lead to mismatches between the RNA and DNA sequences (Cozzuto *et al.* 2020; Jenjaroenpun *et al.* 2021; Liu *et al.* 2021; Abebe *et al.* 2022). Here, for each sample, we calculated the proportion of mismatches, the same results were obtained by our calculation (see *Materials and Methods*) and those from EpiNano (Liu *et al.* 2021). Figure 3a shows the RNA–DNA mismatches for the samples with different types and quantities of modifications. In some samples, such as those with m1A and m6A, the error patterns are similar to the canonical-only samples, even in the samples with high proportions of modified m1A and m6A. But other samples, particularly the samples with biotinylated cytidines and Ys, show a dose-dependent increase in the mismatch rates. In those samples, most of the increases are U-to-X errors (Fig. 3a). We first thought that the U-to-X errors are due to misreading of the Y but then the samples with biotinylated cytidine also have the most U-to-X mismatches.

In addition to sequence mismatches, some of the samples with modified bases have more insertions and deletions than the samples with only canonical bases (Fig. 3b). The modified cytidines (biotinylated-C, hm5C, and m5C) and Y show a dose-dependent increase in insertions and deletions. That modified cytidine nucleotides led to more insertions and deletions may reflect the fact that runs of cytidines in the RNA transcript being analyzed here make up most (40%) of the homopolymer runs of length of at least 3.

After examining the general features, we asked whether each modification gives a distinct pattern of sequence mismatches between the RNA and DNA. The transcript-wide data in Fig. 3a did not reveal distinctive patterns for the different modifications. Since the baseline error rate is high (>10%) as observed in the canonical-only samples, the transcript-wide signals may not be clear given the high background error rate. Therefore, we normalized the error rates of the samples with modified bases to that in the canonical samples, and then we looked at the bases with the most errors to focus on those where the signals should be the highest. Figure 3c shows that for each sample, the 100 bases with the highest error rates are indeed enriched for the modified bases added to the in vitro synthesis of the RNA. In the samples with Y, 84 of the 100 bases with the highest error rates were uridines, while none of the 100 bases with the lowest error rates were uridines. We carried out a similar analysis using Tombo (compare option; Stoiber *et al.* 2017) to predict which bases in the samples were most likely modified, and the results also show an enrichment of cytosines in the top-scoring sites for samples with hm5C and m5C, as well as an enrichment of adenosines in the samples with m6A, but not for the m1A or Y samples (Fig. 3d).

In Oxford Nanopore sequencing, 5 nucleotides occupy the reader-head at 1 time. To examine how neighboring nucleotides influence the identification of modified bases, we focused on the 5-nucleotide windows. In each sample, for each of the 100 sites with the most errors, we looked for enriched motifs in those nucleotides and 2 bases upstream and downstream. The RNA transcripts in this study are synthesized by T7 RNA polymerase, we do not expect the polymerase to incorporate the modified nucleotides in some sequences more than others which contrasts with in vivo RNA modifications that are installed by enzymes such as METTL3/METTL14 that have sequence preferences (Liu *et al.* 2014). In the samples with m6A, we identified an enrichment of adenosines in the neighboring sites. But in the samples with Y, there was only the central U with no enrichment of neighboring nucleotides, suggesting that the Y alone contributes to the error pattern (Fig. 3e). For the other modified bases, no motif enrichment was found.

We further examined whether the modified nucleotides transit through the pores at a different speed by studying how long the nucleotides dwell in the pores. For each sample, we compared the dwell time of the base (as a 5-mer with the base of interest in the middle) in the samples with and without modified bases. We found that all 6 nucleotides with modified bases have highly significantly different ($P\ll 10^{-4}$) dwell times than their canonical counterpart (Table 3). Among them, 5 modifications (m6A, biotinylated C, hm5C, m5C, and pseudoU) led to longer dwell time. Biotinylated C led to a 54% longer dwell time than its unmodified counterpart. For this analysis, we used the 1:2 modified to canonical samples so only a third of the bases are modified assuming equal incorporation of modified and canonical nucleotides by the T7 RNA polymerase. Since we do not know which base has the modification, we assumed that they all do, although they are not all modified. Therefore, the differences in dwell time between the modified base and canonical base identified here are most likely an underestimate; still, we find highly significant differences. Thus, the modifications influence the time it takes for the nucleotides to transit the pores.

**Table 3.** Dwell times of modified nucleotides and their corresponding canonical nucleotides.

Sample	Conc.	Count	Mean (ms)	SD	*P*-value (*t*-test)
m1A	1:2	1,394,248	16.4	25.7	3.12 × 10^−73^
A		685,581	17.1	26.7	
m6A	1:2	2,204,409	17.0	26.9	1.44 × 10^−86^
A		2,170,594	16.5	25.8	
biotin-C	1:2	12,389	23.1	36.7	0
C		2,692,199	15.0	23.4	
hm5C	1:2	239,031	15.9	23.8	1.97 × 10^−49^
C		253,897	14.9	23.2	
m5C	1:2	248,840	18.4	26.9	6.20 × 10^−124^
C		537,692	16.9	25.6	
pseudoU	1:2	2,102,195	17.1	28.6	≪ 10^−125^
U		2,605,240	15.4	25.6	

Conc, ratio of modified nucleotide:canonical nucleotide; SD, standard deviation.

Together, as a group, the nucleotides with nucleobase modifications are distinguishable from the canonical bases when the modification is abundant in the samples. Among the analysis of samples with 1 part modified nucleotides and 2 parts canonical nucleotides, the simple comparison of the RNA sequences to the underlying DNA sequences identified more errors at the adenosines in the samples with modified adenosines (m1A and m6A), the cytidines in the samples with modified cytidines (biotinylated C, hm5C, and m5C) and the uridines in the Y samples. By motif analysis, we found that neighboring adenosines contributed to errors in the m6A samples but not in the m1A samples, this may allow for the 2 modifications to be distinguished. There is no such combination of features that allows us to tell apart the 3 modified cytidines. While all 6 modified bases led to significantly different dwell times in the pores, dwell time alone does not tell apart the different modifications.

### Analysis of the RNA sequences synthesized with sugar modifications

Using the same DNA template, we included 2′-*O*-methylated sugars (Am, Cm, Gm, and Um) at different proportions in the IVT reactions. The samples are deeply sequenced (Table 4), and their PHRED scores are 18 (median scores for 1:2 Am, Cm, Gm, and Um).

**Table 4.** RNA transcripts and their sequence coverage: RNA with modified sugars.

Proportion	Am	Cm	Gm	Um
1:100	8,161	521	13,115	12,104
1:10	10,678	428	8,974	7,347
1:5	12,028	265	9,204	7,478
1:2	10,865	676	10,803	8,163
100%	4,610	302	24	6,999

**Fig. 4.**
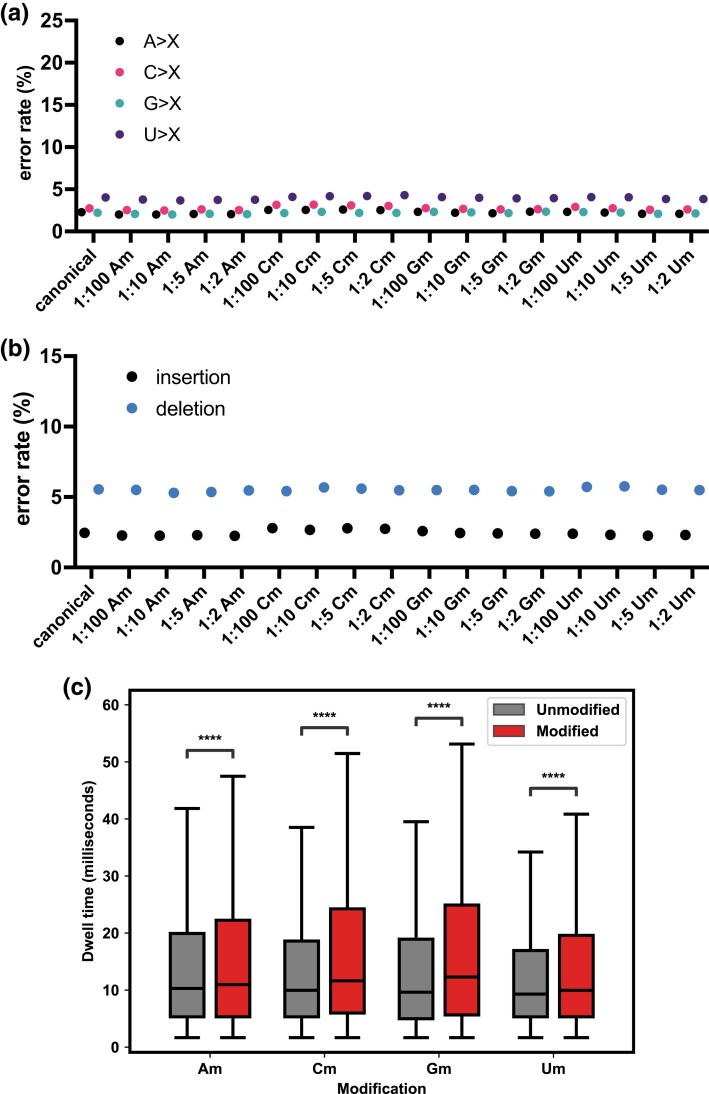
Sequence mismatches, insertions, and deletions in RNA sequences of transcripts synthesized with 2′-*O*-methylated sugar modifications. a) Mismatches between DNA and RNA sequences as in Fig. 3a but for samples synthesized with different 2′-*O*-methylated sugars in different proportions, each sample is shown as a dot. b) Insertion and deletion rates, as in Fig. 3b. c) Dwell times for the 5-mers in the pores with the nucleotide with 2′-*O*-methylated sugar in the center compared with the corresponding 5-mers with only canonical nucleotides, **** denotes *P*-values ≪10^−10^ (Student's *t*-tests).

We carried out the same analyses as above to assess whether the different methods could identify the nucleotides with sugar modifications. Overall, the sugar modifications are more subtle and do not lead to clear patterns that distinguish them from the canonical nucleotides. Figure 4a shows the transcript-wide patterns; we did not identify a sequence mismatch pattern that distinguishes any of the sugar modifications from each other or the canonical sugar. Similarly, Fig. 4b shows no significant differences in the number of insertions and deletions in the samples with sugar modifications compared to those with the canonical nucleotides. Thus, transcript-wide sequence mismatches and the number of insertions/deletions do not distinguish sugar modifications from each other or the canonical bases.

As with the RNA transcripts with base modifications, for the RNAs transcribed in the presence of 2′-*O*-methylated NTPs, we examined the nucleotides with the most errors. We also did not observe a distinguishable error pattern for any of the samples with 2′-*O*-methylated sugars. We assessed the 2 bases upstream and downstream of the bases, and no specific motif was found for any of the samples. Since it has been reported that T7 RNA polymerase does not favor the incorporation of sugar modifications (Ibach *et al.* 2013), it is possible that too few modified sugars were incorporated into the transcripts. To assess whether this may explain why we did not find differences in the RNA sequences with modified sugars and the ones with only canonical nucleotides, we synthesized a set of samples with a 2′-*O*-methyl-NTP and the 3 remaining canonical nucleotides (Table 4). By error analysis and Tombo compare, we found that the sites with the most errors are indeed enriched for adenosines and uridines in the sample with all Am and all Um, respectively (Tables 5 and 6). No such enrichment was identified for the Cm and Gm samples. We also examined the dwell times of the nucleotides with sugar modifications. All 4 sugar modifications led to significantly longer dwell times in the pores, Am, Cm, Gm, and Um dwelled for 7, 20, 25, and 11% longer in the pores than their canonical counterpart, respectively (Fig. 4c).

**Table 5.** Counts of each of the 4 nucleotides in the 100 sites with the least and most errors (as determined by error analysis) among the samples with 2′-*O*-methylated sugars.

Modification (100%)	Bottom 100 #A	Bottom 100 #C	Bottom 100 #G	Bottom 100 #U	Top 100 #A	Top 100 #C	Top 100 #G	Top 100 #U
Am	0	43	48	9	53	15	16	16
Cm	5	21	65	9	49	14	14	23
Gm	17	30	31	22	33	13	26	28
Um	27	35	38	0	8	21	20	51

For example, 53 of the 100 sites with the most errors in the Am samples are A but were read as C, G, or U; thus, they are A-to-X errors.

**Table 6.** Counts of each of the 4 nucleotides in the 100 sites with the least and most errors as determined by Tombo for the samples with 2′-*O*-methylated sugars.

Modification (100%)	Bottom 100 #A	Bottom 100 #C	Bottom 100 #G	Bottom 100 #U	Top 100 #A	Top 100 #C	Top 100 #G	Top 100 #U
Am	7	36	36	21	44	26	23	7
Cm	15	19	41	25	32	26	32	10
Gm	14	32	31	23	31	23	39	7
Um	29	29	33	9	20	21	30	29

### Some of the 10 modifications are distinguishable from each other

**Fig. 5.**
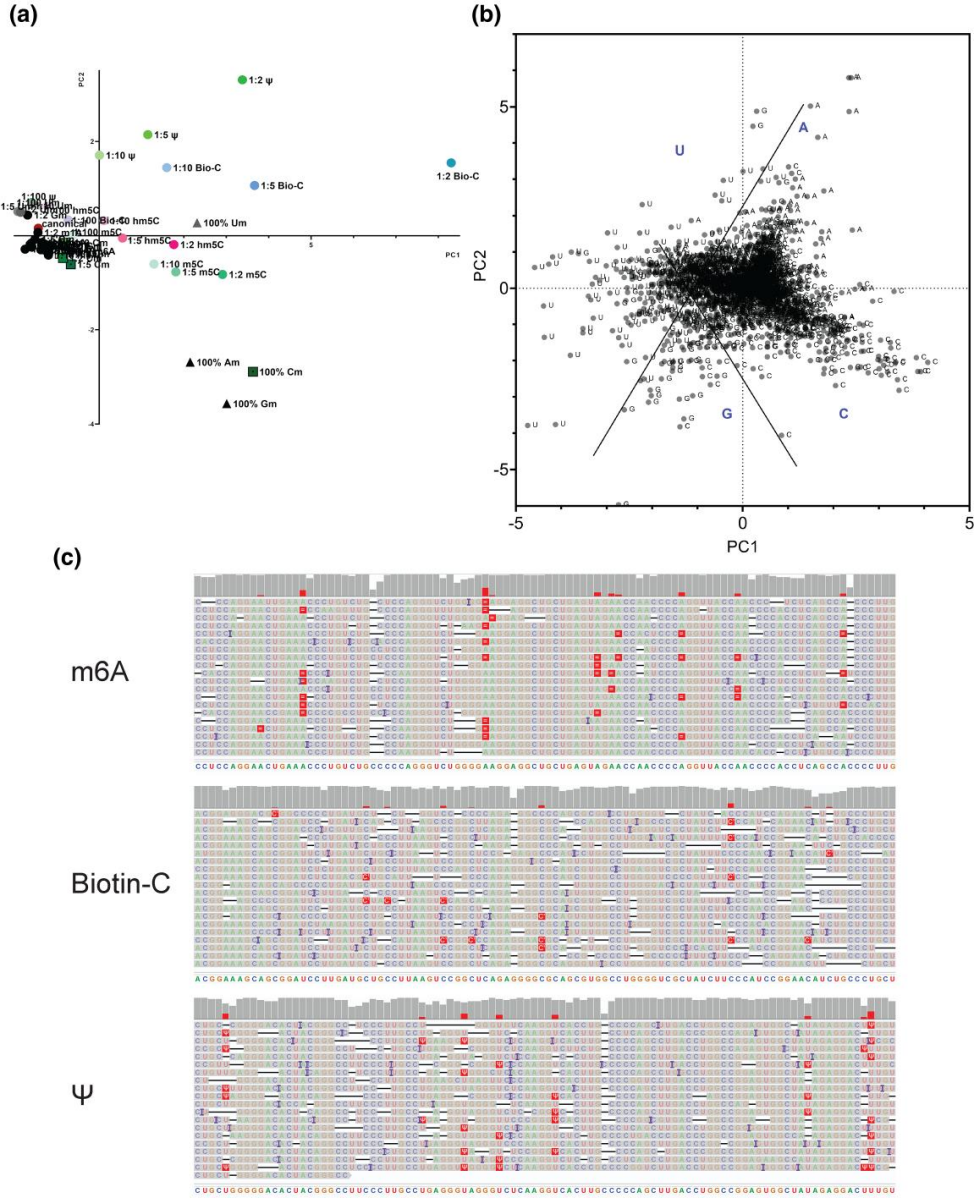
Principal component analysis distinguishes some of the modifications at the transcript and nucleotide levels. PC scores from PCA with a) transcript-wide sequence mismatches (error rates), insertion rate, deletion rate, and dwell times as variables, the first 2 principal components explained 95% of the variance, b) with the same variables as in a) but for each nucleotide in the sample synthesized with biotinylated-C (1 part biotinylated C:2 parts canonical C), the first 2 principle components explained 45% of the variance, the 2 lines are drawn as visual aids to see the clusters for A, C, G, and U. c) using the PCA as in b) along with the sequence errors and dwell time of each sequence read, we identified the sites that are most likely to be modified and annotated them. Data are shown for the RNA synthesized with m6A, biotinylated-C, and Y. Each panel shows sections of 20 sequence reads.

Lastly, we looked at all the samples with only canonical nucleotides and the 10 modifications together. We carried out unsupervised machine learning with 7 variables (error rates of A-to-X, C-to-X, G-to-X, and U-to-X; insertion rate; deletion rate; and dwell time). Figure 5a shows the results of the PCA. PC1 and PC2 captured 65 and 19% of the total variance, respectively. Several of the samples with low (1%) to high (33%) concentrations of modifications are distinct, these include RNA with Y, biotinylated cytidines, 5-hydroxylcytidine, and 5-methylcytidine.

We also carried out PCA with sequence reads to ask whether we can distinguish them. We used a similar concept in population genetic studies where individuals are grouped by populations based on their genotypes of genetic markers that have different allele frequencies across populations (Patterson *et al.* 2006; Price *et al.* 2006). Using the information on the 5-mers from all the samples in ANOVA, we identified the current and dwell times that are most variable (top 5%) across the modifications relative to within each modification. Those current signals and dwell times were then used as variables in the PCA. The results show that PC1 and PC2 captured only 30 and 2.5% of the total variance. The reads are mostly clustered together, except there are a small number of reads from samples with Y or biotinylated C that are outliers (Supplementary Fig. 1). We used different thresholds for the inclusion of variables in the analysis and none of them allowed us to distinguish the reads by modifications.

We next asked whether PCA with data at nucleotide-level would allow us to identify the modified bases and therefore obtain transcript sequences with modifications. Figure 5b is a graph of the PC scores which shows that nucleotides A, C, G, and U are mostly clustered distinctly. For each sample, we then identified the outliers (see *Materials and Methods*) and at those positions, we annotated the sequence reads with errors or longer dwell time as modified nucleotides (Fig. 5c). We used the information from our experimental setup to guide the notations, that is, for the m6A samples, we only looked at the outliers among the As, and for the biotinylated-C samples, we only examined the outliers among the Cs. While we can annotate the most likely modified bases, we had to rely on a priori knowledge which is not possible if we are sequencing RNA from a biological sample.

Together, while it is possible to distinguish the canonical and some modified nucleotides, direct RNA sequencing is not yet able to capture the full complexity of RNA. The sequence reads of the RNAs with different modifications generated here will add to the resources needed to develop base-calling algorithms.

## Discussion

In this study, we used a long (∼2 kb) DNA template for IVT with 10 different modified nucleotides in a range of concentrations to generate RNA transcripts with modified bases or sugars. We also included RNA transcripts with only canonical nucleotides. The incorporation of the modified nucleotides was confirmed by chemiluminescence assay and mass spectrometry analyses. The RNAs were then sequenced on a Nanopore MinION with deep coverage, most samples having >5,000 reads. These are available for benchmarking algorithms for base-calling.

We generated independently 10 sets of RNA with only canonical nucleotides for comparison with their counterparts with modified ribonucleotides. Direct RNA sequencing of the samples with only canonical nucleotides revealed that the error rate of Nanopore RNA sequencing is still >10%.

Our analyses show that, while some modifications can be detected with existing algorithms such as Tombo and analysis of error patterns, we are far from sequencing RNA with all modifications. To obtain the complete sequences with all modifications, several technological advancements are needed. These include reducing the error rates to <0.1%, improving the resolution from 5-nucleotide to single-nucleotide, and developing base-callers that identify the canonical bases, known modifications, and yet-to-be discovered modifications. Given the large number of ribonucleotides per cell, even if we assume 10 pg of RNA per cell, there are >10 billion ribonucleotides in each cell, and thus, even a low error rate of 0.1% has many errors. Improvement in resolution to the single-nucleotide level will facilitate base-calling. Most probably, many modifications are transient and occur at a low level, so they are difficult to detect, and therefore, technical barriers such as poor resolution need to be improved. The biggest challenge of direct RNA sequencing is base-calling. Even if the instrument can capture current signals of a single nucleotide or uses methods such as exo-seq (Wang *et al.* 2022), a very large number of RNAs with different modifications in various sequence contexts need to be synthesized to train and benchmark algorithms. A possible starting point is to identify all modifications in the RNA of human cells by mass spectrometry, synthesize the modified nucleotides, and then use those modified nucleotides in different combinations and concentrations to generate RNAs as training sets. We find it helpful to have independent sets of RNA with only canonical nucleotides for comparison and would recommend the inclusion of RNA with no modifications for controls until the sequencing platforms are fully developed.

RNA-sequencing technologies that provide the complete sequences with all the modifications, at affordable cost, will transform RNA biology and clinical medicine. The complete sequences will elucidate the basic aspects of RNA from splicing to stability. The sequences are crucial for the development of effective RNA-based therapeutics. The exact sequences are necessary for targeting specific RNA, replacing dysfunctional ones and in manufacturing vaccines. Development of sequencing technologies, coupled with analysis methods, is a necessary breakthrough to advance RNA biology and therapeutics.

## Supplementary Material

jkad200_Supplementary_Data

## Data Availability

All Nanopore sequence data are available at NCBI with the accession number: PRJNA976752. The code used in the analysis can be found at https://github.com/vivian-cheung-lab/nanopore_mods. Supplemental material available at G3 online.
