# Supplementary material for: Nanopore-based direct sequencing of RNA transcripts with 10 different modified nucleotides reveals gaps in existing technology
Source: G3 (Bethesda). 2023 Sep 1;13(11):jkad200. doi: 10.1093/g3journal/jkad200 (PMC10627276; doi:10.1093/g3journal/jkad200)
Supplement: jkad200_Supplementary_Data [file jkad200_supplementary_data.pdf]

Supplemental Information

Nanopore-based direct sequencing of RNA transcripts with ten different modified nucleotides reveals gaps in existing technology.

Joshua T. Burdick, Annelise Comai, Alan Bruzel, Guangxin Sun, Peter C. Dedon, Vivian G. Cheung

Figure S1

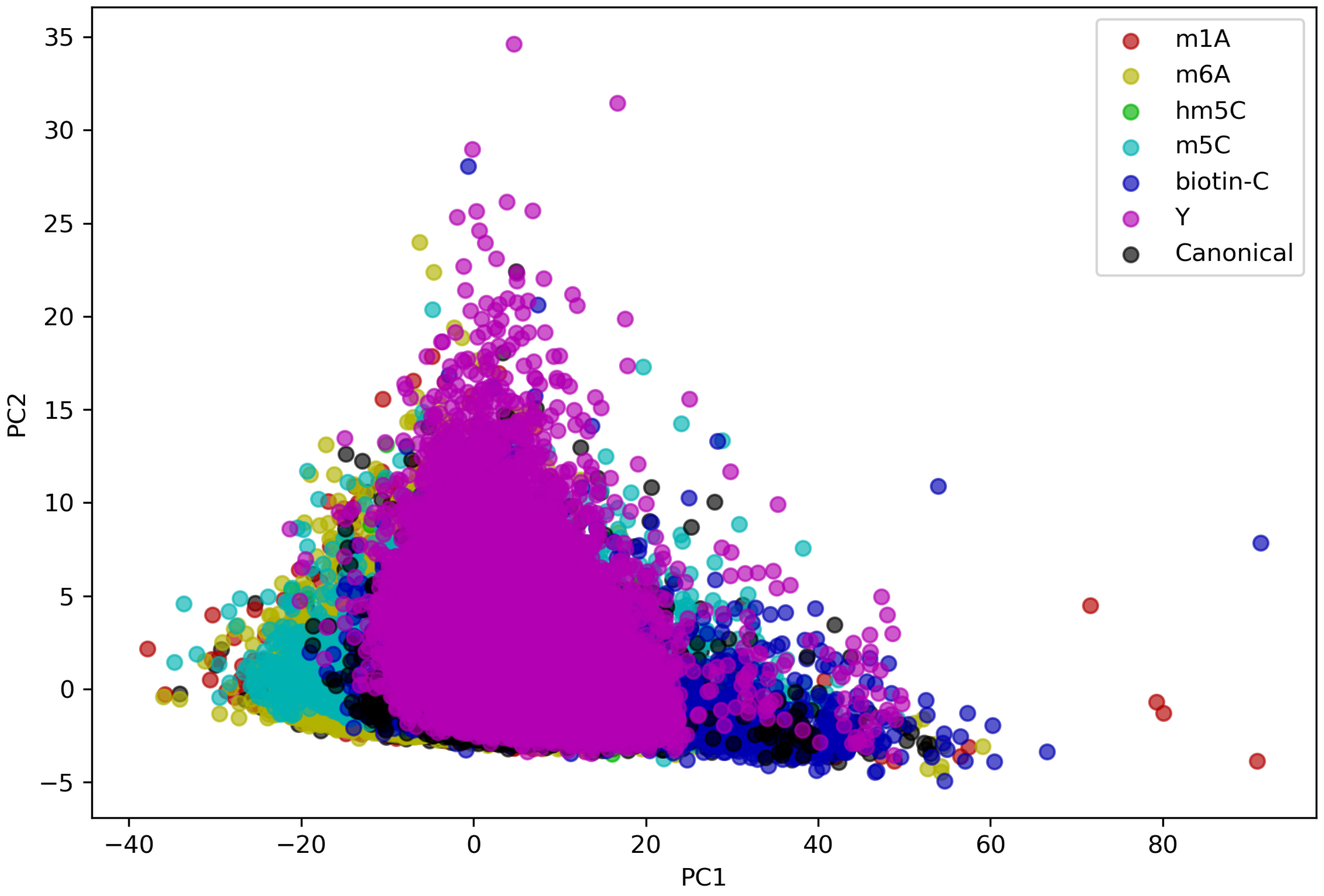

Figure S1. Principal component analysis of sequence reads.

Principal component analysis was performed with current signals and dwell times of modification-informative 5-mers as variables and the sequence reads as samples (see Methods). Scores from PC1 and PC2 which explained 32% of the variance are plotted.

Table S1. HPLC buffer gradient used in the LC-MS/MS quantification of modified ribonucleosides in the IVT transcripts.

**Time (min)**	**Buffer A (%)**	**Buffer B (%)**	**Flow (ml/min)**
0	100	0	0.3
5	99	1	0.3
6	98	2	0.3
7	97	3	0.3
8	95	5	0.3
9	93	7	0.3
10	90	10	0.3
12	88	12	0.3
13	85	15	0.3
15	80	20	0.3
16	25	75	0.3
17	0	100	0.3
18	0	100	0.3
20	0	100	0.3
21	100	0	0.3
25	100	0	0.3

Table S2. Data corresponding to Figure 1B. Quantification by LC-MS/MS of modified ribonucleosides m1A, m6A, hm5C, and Y in the transcripts, for two different concentrations of each ribonucleoside triphosphate added to the IVT reaction. Data represent mean ± SD for three technical replicates

Normalized peak area									
Samples	unmodified	1 Y:10 UTP	1 Y:100 UTP	1 m1A:10 ATP	1 m1A:100 ATP	1 m6A:10 ATP	1 m6A:100 ATP	1 hm5C:10 CTP	1 hm5C:100 CTP
hm^5C	0.00	0.00	0.00	0.00	0.00	0.00	0.00	26045±1059	3204±237
m^1A	3.3±2.1	4.0±0.4	2.7±0.3	33067±3172	8106±1410	4.2±1.2	3.1±0.2	2.4±0.7	2.4±0.6
m^2A	0.00	0.00	0.00	0.00	0.00	15.81	0.00	0.00	0.00
m^6A	0.00	0.00	0.00	14067±893	1767±135	94926±8342	21841±910	0.00	0.00
Y	0.00	992±124	102±12	0.00	0.00	0.00	0.00	0.00	0.00

Table S3. General characteristics of the sequences

Sample (modified:canonical)	Mapped reads	Map %	Read length (ave)	Read length (max)	Phred (median)
canonical only (seq w/m1A)	3066	100%	1235	3919	20
canonical only (seq w/m6A)	8863	100%	1340	6797	15
canonical only (seq w/ biotinC)	9424	100%	1059	4326	17
canonical only (seq w/ 5mC)	1610	100%	1212	2064	17
canonical only (seq w/ hm5C)	794	100%	1142	3307	17
canonical only (seq w/ Y)	9233	100%	1421	5780	18
canonical only (seq w/Am)	6916	100%	1102	3839	18
canonical only (seq w/Cm)	255	100%	1276	2626	17
canonical only (seq w/ Gm)	7907	100%	1169	4696	18
canonical only (seq w/Um)	7415	100%	1468	5920	18
m1A (1:100)	6737	100%	1212	3975	18
m1A (1:10)	7037	100%	1231	3942	18
m1A (1:5)	7689	100%	1188	3908	18
m1A (1:2)	7213	100%	1075	3916	18
m6A (1:100)	11332	100%	1402	5263	18
m6A (1:10)	8068	100%	1421	5276	19
m6A (1:5)	10481	100%	1429	5842	18
m6A (1:2)	8851	100%	1394	4164	18
biotin C (1:100)	1186	100%	999	2317	16
biotin C (1:10)	7691	100%	837	2484	15
biotin C (1:5)	343	100%	846	1955	15
biotin C (1:2)	573	100%	667	1909	15
hm5C (1:100)	306	100%	1119	2126	17
hm5C (1:10)	589	100%	1337	3306	16
hm5C (1:5)	501	100%	1161	2207	17
hm5C (1:2)	720	100%	1232	3426	16
m5C (1:100)	1035	100%	1291	3121	17
m5C (1:10)	2645	100%	1231	2749	16
m5C (1:5)	960	100%	1185	3442	16
m5C (1:2)	732	100%	1283	2060	15
Y (1:100)	7799	100%	1324	3949	18
Y (1:10)	8253	100%	1311	3934	17
Y (1:5)	8108	100%	1390	3923	18
Y (1:2)	7813	100%	1507	4161	15
Y (100%)	452	100%	1618	3698	11
Am (1:10)	10664	100%	1247	4032	19
Am (1:5)	11999	100%	1249	3956	18
Am (1:2)	10845	100%	1247	3965	18
Am (100%)	4516	100%	519	2164	16
Cm (1:100)	520	100%	1199	2701	18
Cm (1:10)	425	100%	1248	2152	18
Cm (1:5)	264	100%	1166	2029	17
Cm (1:2)	672	100%	1191	2142	18
Cm (all Cm)	294	100%	480	1276	15
Gm (1:100)	13078	100%	1186	4010	18
Gm (1:10)	8958	100%	1295	3959	18
Gm (1:5)	9181	100%	1358	3967	18
Gm (1:2)	10779	100%	1365	5803	18
Gm (all Gm)	24	100%	469	1003	15
Um (1:100)	12067	100%	1375	4299	18
Um (1:10)	7324	100%	1340	4014	18
Um (1:5)	7463	100%	1328	4946	18
Um (1:2)	8145	100%	1367	4030	18
Um (all Um)	6465	100%	482	2498	15
